# Spontaneous hemorrhage in adrenal myelolipoma treated with elective laparoscopic adrenalectomy following selective arterial embolization

**DOI:** 10.1002/iju5.12821

**Published:** 2024-12-15

**Authors:** Itsuho Ito, Kosuke Takehara, Yuya Miyazaki, Ayaka Tsuchiyama, Yuta Mukae, Koichi Hayakawa, Ichiro Sakamoto, Junji Irie, Junichi Watanabe

**Affiliations:** ^1^ Department of Urology Nagasaki Harbor Medical Center Nagasaki Japan; ^2^ Coordination Office for Emergency Medicine and International Response, Acute and Critical Care Center, Nagasaki University Hospital Nagasaki Japan; ^3^ Department of Emergency Medicine Nagasaki Harbor Medical Center Nagasaki Japan; ^4^ Department of Radiology Nagasaki Harbor Medical Center Nagasaki Japan; ^5^ Department of Pathology Nagasaki Harbor Medical Center Nagasaki Japan

**Keywords:** adrenal myelolipoma, arterial embolization, hemorrhage, laparoscopic adrenalectomy

## Abstract

**Introduction:**

Adrenal myelolipoma is a benign adrenal tumor that is typically asymptomatic and is rarely associated with hemorrhage or rupture. Here, we present a case of adrenal myelolipoma with spontaneous hemorrhage.

**Case presentation:**

A 72‐year‐old man with a history of obesity and hypertension visited the Department of Emergency Medicine with a sudden onset of severe left flank pain. Enhanced computed tomography showed a left adrenal tumor containing a fat component with a focus of contrast medium visualized extravasation. The patient was diagnosed with adrenal myelolipoma with spontaneous hemorrhage. Selective adrenal arterial embolization was performed to manage the severe pain, and the condition immediately improved. Four months later, laparoscopic left adrenalectomy was performed via a transperitoneal approach. Histopathological examination confirmed the diagnosis of adrenal myelolipoma.

**Conclusion:**

Urgent transarterial embolization followed by elective laparoscopic adrenalectomy is a safe and minimally invasive treatment option for managing adrenal myelolipomas with hemorrhage.

**Abbreviations & Acronyms**
AML: adrenal myelolipoma
BMI: body mass index
CT: computed tomography

**Keynote message**

We describe a case of adrenal myelolipoma with spontaneous hemorrhage. The patient was successfully managed with elective laparoscopic adrenalectomy, following selective adrenal arterial embolization. Urgent transarterial embolization followed by elective laparoscopic adrenalectomy is a safe and minimally invasive treatment option for managing adrenal myelolipoma with hemorrhage.

## Introduction

AML is a benign adrenal tumor composed of adipose tissue and bone marrow elements.^1^, ^2^ Most AMLs are asymptomatic and incidentally discovered during radiological imaging. Surgery should be considered for symptomatic patients exhibiting large tumors with evidence of hemorrhage or tumor growth.^1^ We present a case of AML with spontaneous hemorrhage. The incidence of AML with hemorrhage or rupture is exceedingly rare. The patient described herein was successfully managed with elective laparoscopic adrenalectomy, following selective adrenal arterial embolization.

## Case presentation

**Fig. 1:**
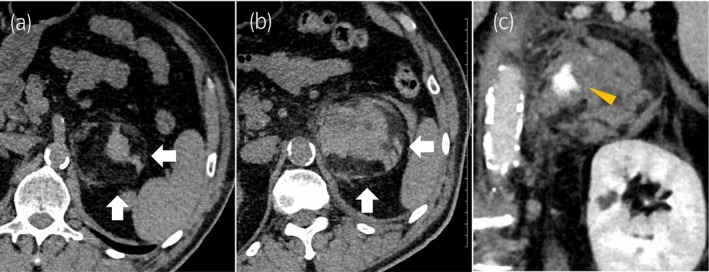
Computed tomography radiographs of the patient. (a) Abdominal CT scan showing a left AML of 68 × 58 mm^2^ before spontaneous hemorrhage (white arrow). (b) Abdominal CT scan showing a left AML with spontaneous hemorrhage of 89 × 83 mm^2^ (white arrow). (c) Abdominal CT with contrast enhancement (coronal image) demonstrated a focus of extravasation (yellow arrowhead).

**Fig. 2:**
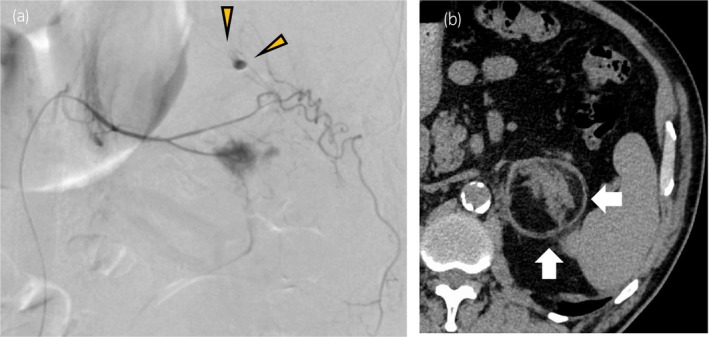
Angiogram and computed tomography radiographs of the patient after catheterization. (a) Selective catheterization and angiogram of the left middle adrenal artery showing extravasation of contrast medium (yellow arrowheads). (b) Four months after embolization, an abdominal CT scan shows a left AML of 58 × 57 mm^2^ (white arrow).

**Fig. 3:**
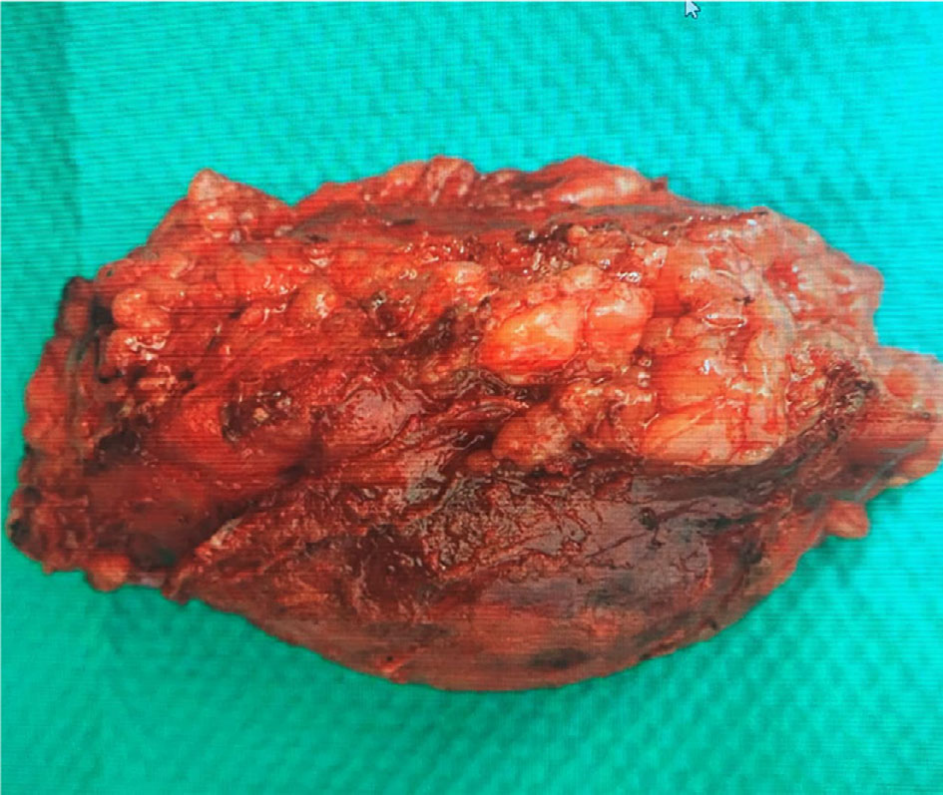
Patient‐derived resected specimen showing an encapsulated yellow solid mass.

**Fig. 4:**
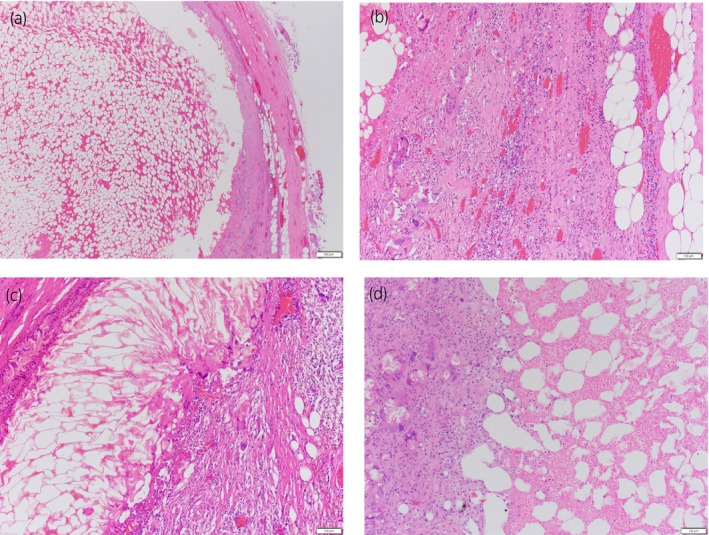
Pathological findings. (a) A low‐power view of the specimen, with fat tissue on the left side and fibrous tissue on the right side (H&E, ×20). (b) Granulomatous multinucleated giant cells are observed on the left side, and compressed, atrophic adrenocortical cells are observed on the right side (H&E, ×100). (c) A lipogranuloma is identified as a multinucleated giant cell tumor (H&E, ×100). (d) Necrotic myeloid tissues are also observed. Multinucleated giant cells are observed on the left side. (H&E, ×100)

A 72‐year‐old man visited the Department of Emergency Medicine, Nagasaki Harbor Medical Center, Japan, with a sudden onset of severe left flank pain. He had a history of hypertension and diabetes mellitus. His BMI was 31 kg/m^2^. The patient had undergone percutaneous cardiac intervention for silent coronary ischemia 3 days before the visit and was receiving dual antiplatelet therapy. During the examination for heart disease, a left AML was incidentally detected on a CT scan, and subsequent hormonal investigations revealed a non‐functional AML (Fig. 1a). He had no history of any trauma. Upon examination, the blood pressure was 205/108 mmHg, and heart rate was 85 bpm. Laboratory results indicated a hemoglobin concentration of 13.5 g/dL and an elevated C‐reactive protein level of 8.58 mg/dL CT revealed a left adrenal tumor with a fat component and hemorrhagic changes. The mass was distinct from the left kidney. Contrast‐enhanced CT showed a hemorrhage in a fatty mass with a focus of contrast medium visualized extravasation. The patient was diagnosed with AML with spontaneous hemorrhage (Fig. 1b,c). Although the hemodynamics stabilized, the patient experienced severe flank pain despite continuous intravenous infusion of fentanyl at increasing doses. Transarterial embolization was performed to control pain. A 4 Fr femoral introducer catheter was placed in the right femoral artery. The left middle adrenal artery from the aorta was selectively catheterized, and angiography demonstrated contrast medium visualized extravasation (Fig. 2a). Selective embolization was performed by injecting N‐butyl‐2‐cyanoacrylate‐lipiodol mixture. Postcontrast injection, no additional extravasation was observed in the embolization area. Angiography of the superior and inferior adrenal arteries revealed no extravasation. Following the procedure, his condition improved rapidly and he was temporarily discharged on the ninth day after embolization. Four months after embolization (Fig. 2b), we performed laparoscopic left adrenalectomy via a transperitoneal approach. Although mild adhesions were present around the tumor, laparoscopic surgery was successfully completed (Fig. 3). The operative time was 232 min, and the estimated blood loss was 30 mL. Histopathological examination confirmed AML with hemorrhage, necrosis, and granulomatous changes (Fig. 4).

## Discussion

AML is a benign adrenal cortical tumor, predominantly composed of adipose tissue and bone marrow elements. The widespread use of imaging techniques has resulted in a remarkable increase in incidentally discovered adrenal masses.^2^ AMLs are commonly occurring adrenal tumors that account for 7%–15% of adrenal incidentalomas.^3^ The pathogenesis of AML is unclear, although it is hypothesized to develop from mesenchymal cell metaplasia or as a result of overstimulation by increased adrenocorticotropic hormone secretion.^1^ These tumors are typically asymptomatic and are most often detected incidentally during abdominal imaging examinations for unrelated symptoms or cancer screening. In the literature, although most AMLs are reported as non‐functional lesions, endocrine dysfunctions occur in 9.5%.^1^ The accepted indications for surgical excision include symptomatic tumors, a size exceeding 4 cm, metabolically active tumors, and a suspicion of malignancy based on an imaging study.^2^ Laparoscopic adrenalectomies have been introduced for AML management, even in the case of giant masses and obese patients.^4^

Hemorrhage or rupture is exceedingly rare and is predominantly observed in large tumors measuring more than 6–7 cm. In a retrospective study of consecutive patients with myelolipoma and acute hemorrhage or tumor rupture requiring surgical intervention, Hamidi *et al*. reported only three cases (0.9%), all with myelolipomas greater than 6 cm.^1^ Shenoy *et al*. reviewed 24 cases of spontaneous rupture of AMLs and reported that rupture had been observed in tumors as small as 7 cm.^2^ The etiology of spontaneous hemorrhage or rupture remains unclear. Takemoto *et al*. reviewed 11 cases of ruptured AMLs in Japan, all of whom were male, and speculated that obesity and hypertension may be risk factors for spontaneous rupture.^5^ Notably, our patient exhibited similar risk factors and had also been receiving dual antiplatelet therapy, which may have increased the risk of spontaneous hemorrhage.

Retroperitoneal hemorrhage or rupture of AMLs reported in the literature were treated with emergent laparotomy.^6^ Recently, there have been increasing reports of transarterial embolization for adrenal gland hemorrhages, although these have been limited to case reports and small case series owing to their low frequency.^7^, ^8^ Transarterial embolization is a minimally invasive, safe, and effective option for acute bleeding emergencies in the abdomen. Giurazza *et al*. reported 17 cases of adrenal gland hemorrhages managed by catheter‐directed embolization with a high success rate.^7^ There have been three reported cases of AMLs with spontaneous hemorrhages treated with transarterial embolization.^8^, ^9^, ^10^ In two of these cases, adrenalectomy was performed as an elective procedure after embolization.^9^, ^10^ Conversely, if initial conservative treatment stabilizes hemodynamics and symptoms in cases of ruptured AMLs, a conservative approach and elective surgery may be considered one of the treatment options.^11^ In the case described herein, a minimally invasive laparoscopic surgery could have been conducted after allowing for absorption of the hematoma for approximately 3 months.^11^ There is no objective evidence for an optimal waiting period prior to surgery. In our case, laparoscopic surgery was successfully performed 4 months after transarterial embolization. There have been no reported cases of spontaneous hemorrhage in AMLs treated with elective laparoscopic adrenalectomy following selective arterial embolization.

## Conclusion

Management of AML with spontaneous hemorrhage and rupture should be determined on an individual basis. The present study has some limitations because it was a single case report. Nevertheless, treating AMLs with spontaneous hemorrhage by urgent transarterial embolization followed by elective laparoscopic adrenalectomy is a safe and minimally invasive therapeutic option.

## Author contributions

Itsuho Ito: Writing – original draft. Kosuke Takehara: Writing – original draft; writing – review and editing. Yuya Miyazaki: Data curation. Ayaka Tsuchiyama: Data curation. Yuta Mukae: Conceptualization. Koichi Hayakawa: Supervision. Ichiro Sakamoto: Supervision. Junji Irie: Investigation. Junichi Watanabe: Writing – review and editing.

## Conflict of interest

The authors declare no conflicts of interest.

## Approval of the research protocol by an Institutional Review Board

Not applicable.

## Informed consent

Written informed consent for publication was obtained from the patient.

## Registry and the Registration No. of the study/trial

Not applicable.
